# Peripheral Blood B-Cell Subsets Frequency and Distribution and the BSF-2(IL-6) to CSIF:TGIF(IL-10) Ratio as Severity-Associated Signatures in Primary Open-Angle Glaucoma: A Case-Controlled Study

**DOI:** 10.3390/biomedicines12030485

**Published:** 2024-02-21

**Authors:** Entsar R. Mokhtar, Asmaa A. Elmadbouly, Omaima I. Abo Elkheir, Mona Nabeh Mansour, Shahinaz El Attar, Mohamed A. Heiba, Mennatullah N. Mohamed, Heba Elhakeem, Lamia A. Gad, Heba Mahmoud Abdelrahman, Rehab Moustafa Kamel, Hekmat M. El Magdoub, Nadia M. Hamdy, Doaa Aly Abd El-Fattah

**Affiliations:** 1 Clinical Pathology Department, Faculty of Medicine (for Girls), Al-Azhar University, Cairo 11884, Egypt; 2 Community Medicine and Public Health Department, Faculty of Medicine (for Girls), Al-Azhar University, Cairo 11884, Egypt; 3 Ophthalmology Department, Faculty of Medicine (for Girls), Al-Azhar University, Cairo 11884, Egypt; 4 Medical Biochemistry and Molecular Biology Department, Faculty of Medicine (for Girls), Al-Azhar University, Cairo 11884, Egypt; 5 Faculty of Medicine, Alexandria National University, Alexandria 21526, Egypt; 6 Kasr Al-Ainy, Faculty of Medicine, Cairo University, Cairo 11562, Egypt; 7 Biochemistry Department, Faculty of Pharmacy, Misr International University (MIU), Cairo 44971, Egypt; 8 Biochemistry Department, Faculty of Pharmacy, Ain Shams University, Abassia, Cairo 11566, Egypt

**Keywords:** glaucoma, B-cells subsets, BSF-2(IL-6) to CSIF:TGIF(IL-10) ratio, POAG, double-negative B cells, in silico

## Abstract

Although primary open-angle glaucoma (POAG) is a major cause of blindness worldwide, patients’ immune response and its relation to the disease course have not been fully unraveled in terms of analyses of circulating B-cell subsets, as well as the association of these subsets with the severity of POAG clinical features. Subjects and Methods: Flow cytometry was used to determine B-cell subset frequencies from 30 POAG patients grouped by hierarchical cluster analysis or the mean deviation (MD) of the visual field (VF) and correlated with the patients’ clinical and pathological data, as well as with BSF-2(IL-6) and CSIF:TGIF(IL-10), which were quantified in peripheral blood samples of patients and controls by ELISA. Results: The total B-cell frequency was increased in the POAG group in comparison to the control group (n = 30). Frequencies of specific B-cell subsets, such as double-negative (DN) and naïve B-cell subsets, were increased in relation to the severity of the POAG disease. However, the unswitched memory B compartment subset decreased in the POAG group. Other non-typical B-cell subsets such as DN B cells also showed significant changes according to the POAG disease severity course. These differences allow us to identify POAG severity-associated inflammatory clusters in patients with specifically altered B-cell subsets. Finally, ocular parameters, biomarkers of inflammation, and other glaucoma-related or non-clinical scores exhibited correlations with some of these B-cell subpopulations. Conclusion: The severity of the POAG disease course is accompanied by changes in the B-cell subpopulation, namely, DN B cells. Furthermore, the existing relationship of the B-cell subset frequencies with the clinical and the inflammatory parameters BSF-2(IL-6), CSIF:TGIF(IL-10), and the BSF-2(IL-6) to CSIF:TGIF(IL-10) ratio suggests that these B lymphocyte cells could serve as potential molecular bio-markers for assessing POAG disease severity and/or progression.

## 1. Introduction

Glaucoma Epidemiology. Glaucoma is a major cause of irreversible vision loss worldwide [1]. Eyewiki (https://eyewiki.aao.org/Primary_Open-Angle_Glaucoma) (Accessed on 9 November 2023) describes glaucoma as being a “neuropathy” of the optic nerve with progressive loss of the nerve fibers and cell bodies of the retinal ganglion cells. Primary open-angle glaucoma (POAG) pathology is the most prevalent progressive and irreversible glaucoma type showing optic nerve damage, without signs or symptoms. Therefore, it is mandatory to predict an individual’s glaucomatous condition evolution and try to compact its progression to facilitate selecting the appropriate therapeutic strategy [2,3], in an attempt to slow down this condition from progressing to blindness. This could only be achieved via early detection for early management.

Problem Definition. The visual field (VF) test characteristic changes provide information about glaucoma occurrence. However, 25 to 33% of retinal ganglion cells must be lost before producing significant VF abnormalities; therefore, early evidence for POAG progression would be missed when using this test [1]. Intraocular pressure (IOP) (mmHg) is the most significant glaucoma risk factor predictor, and up-till-now it is the only parameter to prove treatment efficacy and to ensure decreased blindness risk with no further POAG progression. Yet, during glaucoma management, 30 to 50% of glaucoma patients have normal IOP mmHg, suggesting the presence of other disease-progression contributing factors to address and if these later factors change during POAG management, then disease-course monitoring [4] is now facilitated.

POAG pathophysiology [5] determines the state-of-art biomarkers residing in the aqueous humor or the trabecular meshwork, as well as the optic nerve and peripheral blood [6]. These molecular markers dictate the extracellular matrix status and define cell-signaling or stress molecules and immune surveillance. Therefore, it is noteworthy to mention the “immunologic component” during the neurodegenerative glaucoma “neuropathy” course as one of the risk factors contributing to the onset and progression of glaucoma. Peripheral blood immune cells may have a role during POAG development and/or progression. “B cells” play an important role in the body’s immune responses [7] via the production of antibodies or immunoglobulins (Ig), antigen presentation to T cells, and secretion of cytokines after differentiation to plasma cells. 

B-cell surfaces express the cluster of differentiation (CD) 27 antigen and the immunoglobulin (Ig) delta (D) by mature B cells. There are four human circulating B-cell subsets in blood: naïve B cells (CD27−IgD+), unswitched memory B cells (CD27+IgD+), switched memory B cells (CD27+IgD−), and finally, double-negative (DN) B cells (CD27−IgD−) [8]. More precisely, mature total B cells express the immunodeficiency, common variable, 3 (CVID3); CD19(CVID3) which is CD19+. Therefore, the four subsets of circulating B cells would be better annotated as naïve B cells (CD19+CD27−IgD+), unswitched memory B cells (CD19+CD27+IgD+), classical switched memory B cells (CD19+CD27+IgD−), and DN B cells (CD19+ CD27−IgD−).

According to BioGPS (http://biogps.org/#goto=genereport&id=930) (Accessed on 9 November 2023) CVID3(CD19) biological processes include B-1 B-cell differentiation, proliferation involved in the immune response, Ig-mediated immune response, the antigen receptor-mediated signaling pathway, regulation of the B-cell receptor signaling pathway, and the regulation of B-cell activation. 

BioGPS (http://biogps.org/#goto=genereport&id=939) (Accessed on 9 November 2023) defines CD27(TNFR) as a member of the tumor necrosis factor (TNF) receptor (TNFR) superfamily; it is a universal memory B-cell marker, considered as a co-stimulatory immune checkpoint. The molecular function of TNFR(CD27) is transmembrane signaling receptor activity, and the biological processes are the cell surface receptor signaling pathway and Ig-mediated immune response.

The membrane-attached Ig:antibody receptor IgD as defined by the BioGPS database (http://biogps.org/#goto=genereport&id=3495) (Accessed on 9 November 2023), when a specific antigen binds, will trigger the expansion and differentiation of B lymphocytes to plasma cells to secret Ig, mediating the humoral immunity effector phase; therefore, these cells can eliminate bound antigens detected. Thence, B cell membrane-bound IgD molecules are non-covalently associated with a heterodimer of CD79A and CD79B. IgD expression starts when B cells are transitioning from immature to mature, where IgM and IgD are expressed together. Mature naïve B cells show an increasing amount of IgD along with a somewhat decreased production of IgM, while immature B cells typically express an increased amount of IgM [9].

Before being stimulated by an antigen, naïve B cells express IgD. IgD downregulation is indicative of isotype-switching [8]. Thus, analyzing circulating B-cell subsets and their possible relationship with POAG clinical features and severity is attempted in the current study.

### Research Hypothesis

Glaucoma has an inflammatory arm in its pathology root cause; hence, biomarkers of inflammation-balance that influence the “glaucoma disease course” are worth studying in a POAG patients’ cohort in relation to circulating B-cell subsets.

The proinflammatory cytokine that stimulates B lymphocytes to produce Ig is B-cell stimulatory factor 2:interleukin-6 BSF-2(IL-6). BSF-2(IL-6), according to a database search, is a multifunctional cytokine http://biogps.org/#goto=genereport&id=3569 (Accessed on 9 November 2023) involved in crucial biological processes of acute phase reactions, acute inflammatory response, leukocyte chemotaxis, the positive regulation of T-cell proliferation, positive regulation of tyrosine phosphorylation of STAT protein, positive regulation of the receptor signaling pathway via JAK-STAT, and the positive regulation of B-cell activation [10]. 

Otherwise, cytokine synthesis inhibitory factor/T-cell growth inhibitory factor/IL-10 CSIF:TGIF(IL-10), via an in silico database search http://biogps.org/#goto=genereport&id=3586 (Accessed on 9 November 2023), is an anti-inflammatory cytokine, and its promoter polymorphism has been previously associated with susceptibility to POAG [11]. Biological processes encountered by CSIF/TGIF/IL-10 per the GeneAtlas U133A from the BioGPS database are the upregulation of endothelial cell proliferation, B-cell apoptotic process, Ig production, chronic inflammation in response to antigen stimuli, downregulation of cytokine production for the immune response, nuclear factor kappa B cells’ (NF*_k_*B) sequestration within the cytoplasm, the downregulation of B-cell proliferation, BSF-2(IL-6), and TNF production. 

The study aimed to determine peripheral blood B-cell subsets’ frequency and distribution in relation to inflammation biomarkers as severity-signatures in a case-controlled POAG study. This is achieved via studying the frequency and distribution of B-cell subsets, the inflammation markers BSF-2(IL-6) and CSIF:TGIF(IL-10), and their ratio in peripheral blood samples from POAG patients’. Second, the study aimed to assess the utility of various B-cell subsets and the BSF-2(IL-6) to CSIF:TGIF(IL-10) ratio as potential molecular markers for POAG disease severity. 

## 2. Subjects and Methods

Study Design. A case-controlled single-center observational study, was carried out during the period from May 2022 to March 2023. 

Sample Size Calculation. We used the Epi Info^TM^ version 7.2 build 7.2.6 25 October 2023 (by the United States Centers for Disease Control and Prevention (CDC)) for sample size calculation public domain software, considering a ratio of controls to cases of 1:1, two-sided confidence level of 95%, and level of significance at 5% for 80% study power. The minimum sample size calculated for this study was 59 cases based on a 4% prevalence of POAG in Africa [12].

Study Participants. Thirty patients with POAG were recruited from the Outpatient Clinic and the Department of Ophthalmology of Al-Zahraa University Hospital, Faculty of Medicine for Girls, Al-Azhar University, Cairo, Egypt. They were compared with 30 age- and sex-matched apparently healthy individuals as the control group, whose selection was based on the absence of the clinical signs of primary or secondary glaucoma or any previous or current eye disease(s). 

Medical and Family History and Examination. Full medical history was recorded for all participants and a comprehensive ophthalmic examination for patients was performed to confirm their eligibility to enroll in the study after signing the informed consent (IC). Examination was conducted by an expert at the Department of Ophthalmology, Al-Zahraa University Hospital, Faculty of Medicine for Girls, Al-Azhar University, Cairo, Egypt, including visual acuity testing for visual clarity or sharpness determination (small letters identification test), a bright-light slit-lamp bio-microscopy exam (BQ-900, Haag-Streit, Köniz, Switzerland) for eye structure examination, non-contact Air-Puff Tonometry (mputeCT-1 Corized Tonometer, Topcon Ltd., Newbury, UK) for IOP (mmHg) measurement, gonioscopy for identifying the open-angle from the closed-angle glaucoma in a dark room using a goniolens (Brand: Madhu; Made In New Delhi, India; Code: MIPL/I3), dilated fundus examination, and the high-resolution micron-scale imaging method which scans the eye tissue optical coherence tomography (OCT) (Humphrey Instruments Ltd., San Leandro, CA, USA) for a small picture target. From the corners, VF testing and analysis is a standard 24-2 test program using the Humphrey VF Analyzer (Carl Zeiss Meditec, Inc., Dublin, CA, USA) that is performed in a semi-dark room with best-corrected near vision correction. The test is repeated if it is found to be non-reliable to detect a learning effect and maximize reliability indices. The mean deviation (MD) values represent the overall mean departure of sensitivity at specific retinal points from the age-corrected normal values, and the pattern standard deviation (PSD) represents focal loss or variability within the field and considering any generalized depression. 

Patients with POAG were diagnosed according to the European Glaucoma Society Terminology and Guidelines for Glaucoma [13]. POAG patients had an IOP (mmHg) elevation (more than 20 mmHg), glaucomatous disc changes in increased cup-to-disc (C/D) ratio, disc notching, and neuro retinal rim thinning, with corresponding glaucomatous changes in the VF and retinal nerve fiber layer (RNFL) thickness by OCT. The severity of glaucoma was determined based on the MD of VF, where the early glaucomatous loss was an MD of ≤6 dB, moderate glaucomatous loss 6 ≤ MD ≤ 12 dB, and severe glaucomatous loss > 12 dB. Patients having closed-angle glaucoma, age-related macular degeneration, high myopia, retinitis pigmentosa, inflammatory diseases, ischemic disease, a history of intracranial lesions, increased IOP (mmHg) due to other known etiologies as trauma, uveitis, or neovascular glaucoma were excluded. Those with the presence of hypertension (HTN), diabetes mellites (D.M), cardiovascular diseases (CVD), cancer, and autoimmune diseases such as rheumatoid arthritis (RA), scleroderma, and lupus syndrome, as well as patients receiving any systemic drugs affecting the immune system, were also excluded from the study. 

Blood Samples Collection. Under controlled aseptic conditions, 5 mL of venous blood was withdrawn from each participant. Each blood sample was divided into two portions, one of 2 mL blood was transferred into serum gel separator tube, centrifuged for serum separation, and the sera obtained were stored at −20 °C for BSF-2(IL-6) and CSIF:TGIF(IL-10) levels’ measurement by ELISA. A total of 3 mL blood was transferred to an EDTA-containing tube, immediately (within 2 h) processed for CBC, and measured to determine the percentage of total B cells (CD19+), DN B cells (CD19+CD27−IgD−), unswitched memory B cells (CD19+CD27+IgD+), naïve B cells (CD19+CD27−IgD+), and classical switched memory B cells (CD19+CD27+IgD−) by flow cytometry (FC). 

Laboratory Investigations. A complete blood count (CBC) was performed by a fully automated hematology analyzer (Sysmex, KX21N, Kobe, Japan). The platelets-to-lymphocytes ratio (PLR), neutrophils-to-lymphocytes ratio (NLR), and monocytes-to-lymphocytes ratio (MLR) were calculated.

B-cell subsets were analyzed by an FC assay using a multi-color Navios EX (Beckman Coulter, Marseille, France) at the Clinical Pathology Department, Al-Zahraa University Hospital. A total of 1 ml of blood sample was washed 3 times by warm saline after adjusting the cell count (1 × 10^6^ peripheral blood mononuclear cells), then 50 μL of that adjusted washed fresh blood sample was added to each of two polystyrene tubes.

The first tube contained 5 μL of each of the following fluorochrome-conjugated antibodies; FITC-conjugated anti-human surface IgD (Lot No. 200026), PE-conjugated anti-human surface CD27 (Lot No. 200045), and APC-conjugated anti-human surface CD19 (Lot No. 200102) (all from Beckman-Coulter, Marsellia, France). The second tube was not loaded by any fluorochrome-conjugated antibodies to determine the auto-florescence. After 15 min incubation at room temperature in the dark, a lysis reagent was added for 8 min for lysing RBCs before cells were washed with phosphate-buffered saline and centrifuged at 500× *g*.

In total, 100,000 events (number of cells counted using flow cytometry) were acquired for analysis to ensure that rare populations in the peripheral blood were evaluated.

**Figure 1:**
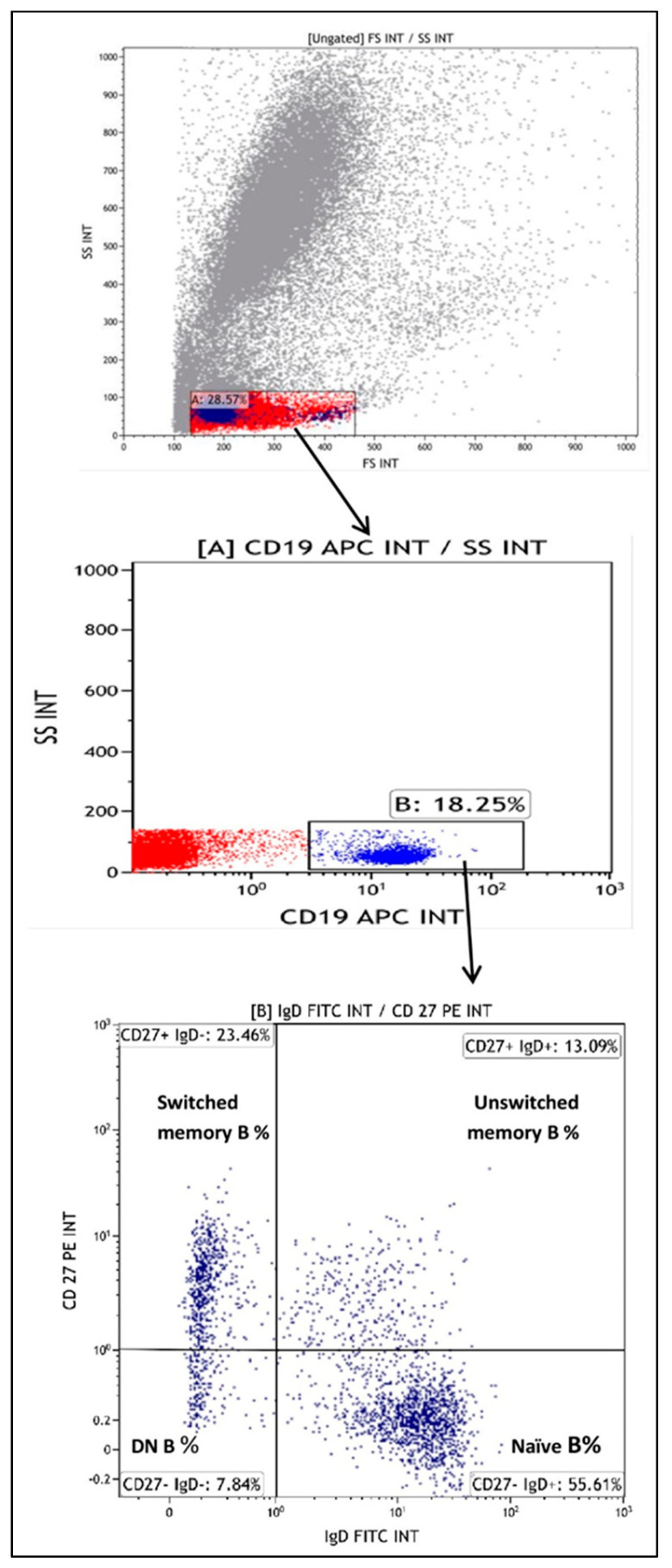
Gating strategy for the detection of B cells and their subsets, where initial gating on mature lymphocytes was performed using dot plot forward and side scatter (**upper graph**), then subsequent gating was taken on mature B cells (CD 19+) using side scatter (SS)/CD19 APC INT (**middle graph**). The quadrant plot using IgD FITC INT (*x*-axis) and CD27 PE INT (*y*-axis) was drawn gated on CD19+ lymphocytes for the determination of B-cell subsets (**bottom graph**).

The gating strategy used dot plot forward and side scatter, with initial gating on mature lymphocytes. Then, another graph was taken for gating on B lymphocytes (CD19+). A quadrant plot was drawn representing anti-IgD on the *x*-axis and CD27 on the *y*-axis which was gated on CD19+ lymphocytes. DN B cells were identified as the CD27−IgD− population, unswitched memory B cells were identified as the CD27+IgD+ population, naïve B cells were identified as the CD27−IgD+ population, and classical switched memory B cells were identified as the CD27+IgD− population. This gating strategy is illustrated in Figure 1.

### 2.1. BSF-2(IL-6) and CSIF:TGIF(IL-10) Assays by ELISA 

Serum BSF-2(IL-6) and CSIF:TGIF(IL-10) levels were measured using the commercially available human ELISA kits, supplied by Bioassay Technology Laboratory (Shanghai, China), Lot No. 202205001, 202205001, and Catalog No. E0090Hu, E0102Hu, respectively. Using an ELISA system, which included a plate shaker–incubator (Thermo-Shaker from EU for Grant Instruments Ltd., Cambs, UK), a plate reader (AS 1851 from DAS, Palombara Sabina, Italy), and an ELISA washer (ELx50 Biokit, Rome, Italy) according to the manufacturer’s instructions, the assays were conducted using serum samples from both controls and patients.

### 2.2. In Silico Database(s) Search and Bioinformatics Analysis

#### In Silico Identification of Immune Cells

To visualize closely related immune cells from the human peripheral blood mononuclear cells, single cells, or from eye immune cells, determination was performed using uniform manifold approximation and projection (UMAP) [14] using the Human Universal Single Cell Hub (HUSCH) which is an scRNA-seq database http://husch.comp-genomics.org/#/info_tissue/ (Accessed on 4 September 2023). 

### 2.3. PICKLE (Protein InteraCtion KnowLedgebasE)

PICKLE (Protein InteraCtion KnowLedgebasE) [15] Release 3.3, 1 October 2021. http://www.pickle.gr/ (Accessed on 9 November 2023). PICKLE is a meta-database for the direct protein–protein interactome of the human proteomes, integrating publicly available source protein–protein interaction (PPI) databases via genetic information ontology. The visualization utilized Cytoscape.js 3.3.0. 

### 2.4. Gene–Gene Interactions and Pathways by Bioinformatics Analysis

Prediction was carried out of the B-cell surface antigens CD19/CVID3 and CD27/TNFR’s top interacting genes via gene-interaction at the University of California Santa Cruz (UCSC) [16] Genome Browser RRID:SCR_005780. Genomics institute http://genome.ucsc.edu/index.html (Accessed on 6 September 2023).

### 2.5. Statistical Analysis

This work used SPSS software v 26.0 (IBM, Armonk, NY, USA) https://www.ibm.com/products/spss-statistics (Accessed on 13 July 2023) for collected data analysis. The testing of groups’ data for normality was conducted using the Shapiro–Wilk normality test. For the non-normally distributed data, the median (interquartile range (IQR) 25th percentile–75th percentile: 1st–3rd quartile) was used. Mann–Whitney (U) was utilized to compare the latter. For qualitative data, dichotomous parameters and the absolute number (percentage) n (%) was the presentation form, and the Chi-square test (*x*^2^) was used for comparison. To find the best cutoff, sensitivities (SNs), specificities (SPs), and the area under the curve (AUC), the receiver operating characteristic (ROC) curve was produced. Finally, the correlation between various variables was assessed using Spearman’s correlation coefficient *r*. The significance level was set at a *p* value of less than 0.05. Cohen’s *q* effect size measure was used to interpret the difference between two correlations with proposed categories for the interpretation: <0.1: no effect; 0.1 to 0.3: small effect; 0.31 to 0.5: intermediate effect; >0.5: large effect [17] using the online calculator https://www.psychometrica.de/effect_size.html (Accessed on 2 February 2024).

## 3. Results

### 3.1. POAG Patients and Controls Demographic Characteristics, Clinical and Laboratory Data Results 

Regarding demographic data (Table 1), POAG patients and healthy controls had no significant difference in terms of age or sex. Visual acuity was significantly lower in glaucoma patients (*p* < 0.001). Other clinical data showed a significant increase in IOP, C/D ratio, MD, and PSD in POAG patients as compared with controls (*p* < 0.001).

There was a significant increase in the absolute monocytic count and MLR in POAG patients when compared to controls (*p* < 0.05).

There was a significant increase in total B cells % in POAG patients in comparison to controls (*p* < 0.001). Upon testing the distribution of different B-cell subsets in POAG patients, there was a highly significant increase in the frequencies of the DN B-cell subset (CD19+CD27−IgD−) and the naïve B-cell subset (CD19+CD27−lgD+) (*p* < 0.001) for all POAG patients, while a significant decrease in the unswitched memory B-cell subset (CD19+CD27+IgD+) (*p* < 0.001) was found in this group as compared with the control group.

POAG patients had significantly increased serum pro-inflammatory BSF-2(IL-6) marker level (*p* < 0.001), decreased anti-inflammatory CSIF:TGIF(IL-10) (*p* < 0.001), and an increase in their ratio (*p* < 0.001).

Moreover, there was a significant positive correlation between the percentage of the DN B-cell subset and the MD of the visual field (marker of the clinical severity of glaucoma) (r = 0.876, *p* < 0.001). However, there was a significant negative correlation between the unswitched memory B-cell subset % and the MD of the visual field (r = −0.838, *p* < 0.001) (Table 2). BSF-2(IL-6), but not the CSIF:TGIF(IL-10), and the BSF-2(IL-6) to CSIF:TGIF(IL-10) ratio were positively correlated with the MD of the visual field (r = 0.684, *p* < 0.001). Furthermore, the DN B-cell subset was the only B-cell subset that showed a significant positive correlation with BSF-2(IL-6) and the BSF-2(IL-6) to CSIF:TGIF(IL-10) ratio (Table 2). It is noteworthy to mention that the effect size calculated for the correlation differences between the significant correlations and the non-significant correlations results for the B-cell subsets or the inflammatory biomarkers supports the Spearman’s correlation *(r)* significant results among the POAG patients’ group (n = 30).

The discriminative utility of B-cell subsets and inflammation biomarkers for POAG patients (n = 30).

**Figure 2:**
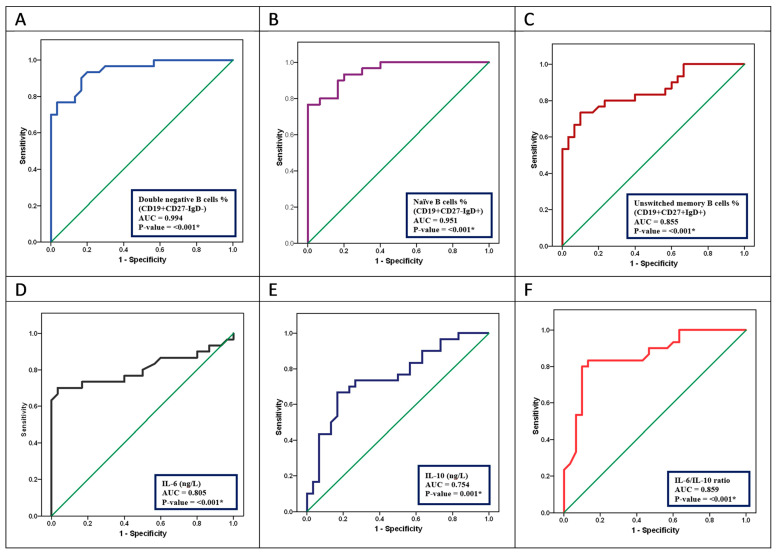
ROC curve analysis showing the AUC for B-cell subsets: (**A**) DN B cells, (**B**) naïve B cells, (**C**) unswitched memory B cells, (**D**) BSF-2(IL-6), (**E**) CSIF:TGIF(IL-10), (**F**) BSF-2(IL-6) to CSIF:TGIF(IL-10) ratio to differentiate POAG patients (n = 30) from healthy controls (n = 30). * Statistical significance *p*-value < 0.05.

ROC curve analysis (Table 3, Figure 2) was performed for B-cell subsets, BSF-2(IL-6), CSIF:TGIF(IL-10), and the BSF-2(IL-6) to CSIF:TGIF(IL-10) ratio to determine the diagnostic performance of these parameters in POAG patients. At the total B-cells cut-off point of >7.6%, POAG patients were identified with a sensitivity of 96.7% and specificity of 100%. At DN B cells cut-off point of >8.15%, POAG patients were identified with a sensitivity of 90.0% and a specificity of 83.3%. At the naïve B-cell subset cut-off point of >44.1%, POAG patients were identified with a sensitivity of 90.0% and a specificity of 83.3%.

At the unswitched memory B-cell subset cut-off point of <14.45%, POAG patients were identified with a sensitivity of 83.3% and a specificity of 90.08%. At the BSF-2(IL-6) cut-off point of >47.0 (ng/L), POAG patients were identified with a sensitivity of 73.3% and a specificity of 83.3%. At the CSIF:TGIF(IL-10) cut-off point of <87.8 (ng/L), POAG patients were identified with a sensitivity of 73.3% and a specificity of 73.3%. At the BSF-2(IL-6) to CSIF:TGIF(IL-10) ratio cut-off point of >0.55, POAG patients were identified with a sensitivity of 80.0% and a specificity of 90.0%.

From ROC analysis, total B and DN B cells exhibited an excellent capability of diagnosing POAG, where DN B cells exhibited a significantly better performance than the BSF-2(IL-6) to CSIF:TGIF(IL-10) ratio in diagnosing POAG; therefore, these can be used as molecular predictors for POAG patients. 

Table 4 shows the distribution of B-cell subsets and inflammation biomarkers according to glaucoma severity in POAG patients’ group (n = 30).

Based on the MD of the visual field, 12 (40%) cases out of 30 cases with POAG had mild-to-moderate glaucoma, while 18 (60%) patients had severe disease (Table 4). Upon comparing both groups, the DN B cells, BSF-2(IL-6), and BSF-2(IL-6) to CSIF:TGIF(IL-10) ratio were significantly increased in severe cases, while unswitched memory B cells were significantly decreased (*p* ≤ 0.001) when compared to mild-to-moderate cases.

### 3.2. In Silico Databases Analysis 

**Figure 3:**
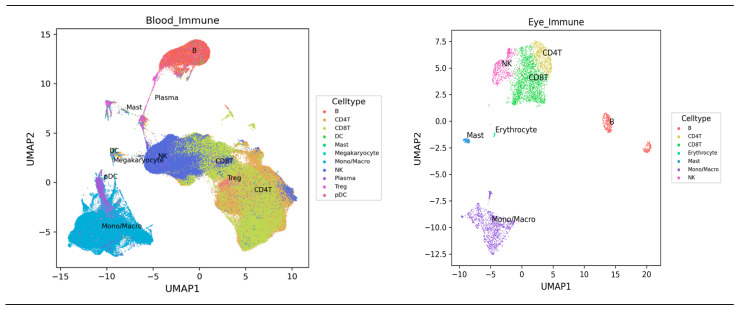
Blood and eye immune cell types expression analysis by the HUSCH http://husch.comp-genomics.org/#/info_tissue/Blood and http://husch.comp-genomics.org/#/info_tissue/Eye, respectively (Accessed on 4 September 2023) [UMAP, uniform manifold approximation and projection].

The results for the identification of immune cells from blood and eye are shown in Figure 3 (Accessed on 4 September 2023) showing blood and eye immune cells’ annotation pattern details of hematopoietic cell clustering (http://husch.comp-genomics.org/#/info_tissue/Blood) and (http://husch.comp-genomics.org/#/info_tissue/Eye) eye immune cells’ annotation details. Cell type: B, with documented markers CVID3(CD19), CD79A, and MS4A1, studied by the human universal single-cell hub (HUSCH) [UMAP, uniform manifold approximation and projection].

**Figure 4:**
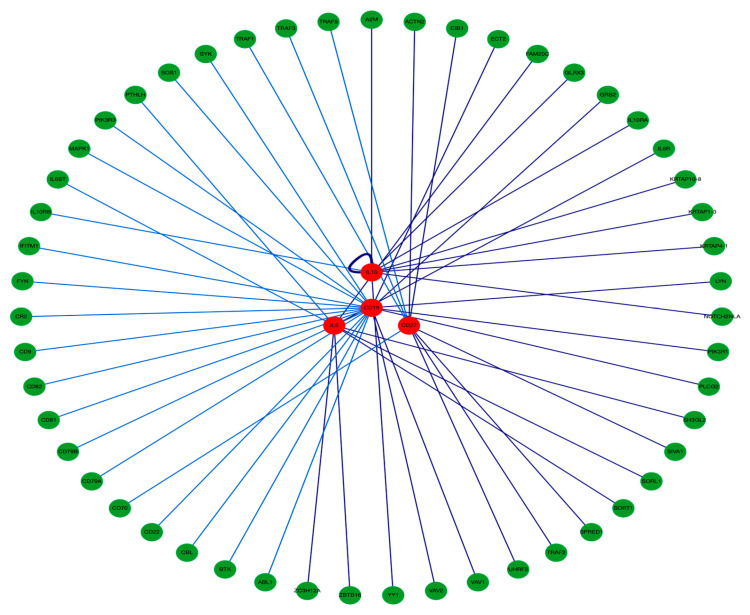
Concentric layout network visualization of the studied B-cell surface CDs CVID3(CD19) and TNFR(CD27) and the inflammatory biomarkers BSF-2(IL-6) and CSIF:TGIF(IL-10) retrieved from PICKLE (with 55 edges and 58 nodes, PPI quality 2, normalization level protein (UniProt), cross-checking (default) filtering method, first neighbors network setup). http://www.pickle.gr/Visualize/Display?ids=4410,5027,3427,4810&normalizationLevel=uniprot&queryType=normal&dataset=crosschecked&org=9606 (Accessed on 9 November 2023).

B-cell surface CDs and the studied inflammatory markers interaction with other genes as well as with each other, are visualized in Figure 4, retrieved from PICKLE database http://www.pickle.gr/Visualize/Display?ids=4410,5027,3427,4810&normalizationLevel=uniprot&queryType=normal&dataset=crosschecked&org=9606 (Accessed on 9 November 2023).

**Figure 5:**
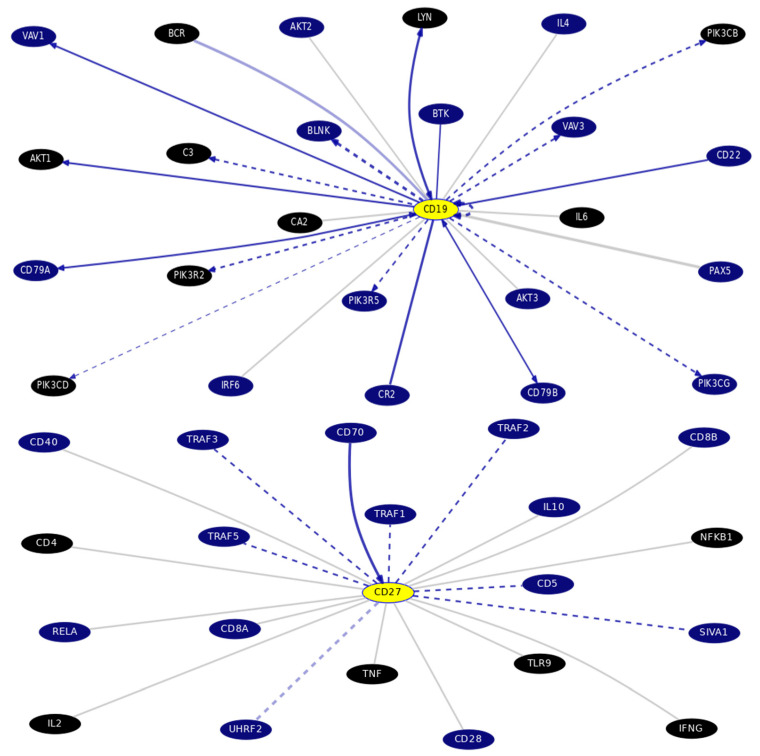
B-cell surface antigens CVID3(CD19) and TNFR(CD27)’s top interacting genes, highlighting the Drug Bank interaction, obtained via gene interactions and pathways, using the UCSC Genome Browser Gene Interaction Graph https://genome.ucsc.edu/cgi-bin/hgGeneGraph?gene=CD19&supportLevel=text&hideIndirect=on&geneCount=25&geneCount=25&geneAnnot=drugbank&1=OK&lastGene=MIR21 and https://genome.ucsc.edu/cgi-bin/hgGeneGraph?gene=CD27&supportLevel=text&hideIndirect=on&geneCount=25&lastGene=MIR21&geneCount=25&geneAnnot=drugbank&1=OK, respectively. Accessed on 6 September 2023. [Black colored genes: treatment hits by Drug Bank; Gray continuous line: no curated information, text mining is evident; Blue continuous line: interaction from several datasets with text mining; Blue dashed line: results displaying dataset, interaction was curated from source document and no text-mining data].

B-cells surface antigens gene–gene interactions and pathways from curated databases and text-mining (Figure 5) (Accessed on 6 September 2023) on the UCSC genomics institute: https://genome.ucsc.edu/cgi-bin/hgGeneGraph?gene=CD19&supportLevel=text&hideIndirect=on&geneCount=25&geneCount=25&geneAnnot=drugbank&1=OK&lastGene=MIR21 and https://genome.ucsc.edu/cgi-bin/hgGeneGraph?gene=CD27&supportLevel=text&hideIndirect=on&geneCount=25&lastGene=MIR21&geneCount=25&geneAnnot=drugbank&1=OK for the B-cell surface antigens CVID3(CD19) and TNFR(CD27), respectively.

The B-cell surface antigen CVID3(CD19)’s top interacting genes are BSF-2(IL-6), targeted by ginseng and anti-cytokines/IL therapy (drug bank). However, the surface antigen TNFR(CD27)’s top interacting genes are CSIF:TGIF(IL-10), where CSIF:TGIF(IL-10) diminishes TNFR (CD27) expression on the B-cell surface (CD27-IL-10). B-cell deletion caused by TNFR(CD27) induced the production of INF gamma in T cells (CD27-INFgamma) treated by glucosamine.

## 4. Discussion

Fortunately, the critical neuroinflammation pathogenic event is treatable in the glaucoma disease [18]. Glaucoma is a complex inflammatory neurodegenerative disorder of the eye and is one of the main causes of irreversible blindness [19].

Lymphocytes constitute one important cellular component of the immune system and are considered active promoters and active regulator players in various inflammatory diseases [20] including glaucoma. Recently, research has focused on the “glaucoma immunological component” where the cytokine-mediating low-grade inflammatory reaction activating the immune response may be crucial in glaucomatous optic neuropathy early development [21].

In the current study, we investigated the frequency of total B cells and the distribution of their subsets in POAG patients’ peripheral blood, where a significant increase in the total B cells % in POAG patients as comparable to the control group was recorded. This was explained by Yu et al., as a result of an excessive B-cell-mediated immune response [7], where the role of B lymphocytes in glaucoma is immune-mediated for glaucoma-induced retinal ganglion cells’ destruction [22]. Moreover, glaucoma patients’ retinas might experience B-cell infiltration [23]. Therefore, our findings come along with these studies addressing B-cell role in glaucoma development.

Upon testing the distribution of different B-cell subsets in a POAG patients’ cohort, there was a highly significant increase in the frequencies of DN B-cell subset (CD19+CD27−IgD−) and naïve B-cell subset (CD19+CD27−lgD+), while a significant decrease was seen in the unswitched memory B-cell subset (CD19+CD27+IgD+). These results depicted naïve B-cell generation enhancement during glaucoma. The lowered percentage of the unswitched memory subset (CD19+CD27+IgD+) could be attributed to its accumulation in germinal centers of secondary lymphatic organs, responsible for persistent Ig:antibodies production. Pre-existing memory B cells might be re-activated and differentiated into atypical late memory B cells known as the DN B-cell subset, which explains the significant increase in this cell population in our POAG patients. DN B cells, named age-associated B cells, and atypical memory B cells both have a dual function, both as pathologic cells mediating low-grade proinflammatory cytokines’ production [24,25] or as protective cells via antigen presentation and antigen-targeted immune responses co-stimulation [26]. These B cells are expanded in the peripheral blood of both elderly healthy individuals and people with chronic infectious diseases, autoimmune disorders [27,28] such as in systemic lupus erythematosus patients, and obesity, diabetes, cardiovascular diseases, and cancer [29]. 

The current study presented an increased level of circulating DN B cells with a positive correlation of this population with the POAG severity index MD of VF. These data suggest that the DN B-cell subset has a pathogenic role in the POAG disease and might be a potential molecular biomarker for monitoring POAG disease progression.

Although class switched memory B cells were reported to show a close relationship with DN B cells in clonal analysis [30], there was no significant difference in the former B-cell subset between the current POAG patients’ group and the control group.

Human blood B cells in different inflammatory environments produce different cytokine profiles [31]. Intense inflammatory staining in the optic nerve head of glaucoma eyes with an elevated proinflammatory BSF-2(IL-6) levels have been witnessed during histological examinations of the human retina [32]. Ulhaq et al., (2021) highlighted the importance of the BSF-2(IL-6) cytokine in the ocular inflammatory process in glaucoma and confirmed its utility as an early marker of injured retinal ganglion cells in glaucomatous animal models [11]. This is in line with our study findings.

Yang et al., (2019) found an alteration in BSF-2(IL-6), but not CSIF:TGIF(IL-10), levels in T-cell culture supernatant from glaucoma patients [33]. In accordance with these results, the Irkec et al., study results showed a low level of CSIF:TGIF(IL-10) production in glaucomatous individuals [34], as was observed in the current study. The reverse was demonstrated in the study by Chua et al. [35]. This discrepancy in the inflammatory biomarkers’ results could be explained on the basis of patients’ different age characteristics and various disease durations and activity, as well as the difference in treatment(s) they received.

CSIF:TGIF(IL-10) is a key regulator of the systemic anti-inflammatory responses and functions to protect glaucoma patients from the persistent low-grade inflammatory state causing eye tissue damage. CSIF:TGIF(IL-10) enhances the survival, proliferation, differentiation and isotype switching of human B cells.

Gramlich et al., reported glaucomatous retinal tissues having dysregulated anti- and pro-inflammatory cytokines patterns of increased TNF-α, IL-1β, BSF-2(IL-6), and IL-8 levels [36] as compared to the non-glaucoma controls. Moreover, a notable production of CSIF:TGIF(IL-10) was observed which downregulates “unnecessary” immune responses [37]. 

Borkenstein et al. and Takai et al., found lower BSF-2(IL-6) levels in eyes from POAG patients. This discrepancy in BSF-2(IL-6) results may be due to variations in disease severity or variations in the patients’ degree of systemic inflammation encountered [38,39].

In the present study, there was a significant positive correlation between both BSF-2(IL-6) and the BSF-2(IL-6) to CSIF:TGIF(IL-10) ratio with the MD of the VF, which is in line with the circulating levels of both in patients’ blood. Similar results were obtained by Ulhaq et al., 2021 [11], demonstrating the imbalance of the systemic inflammatory response as marked by an increased BSF-2(IL-6) to CSIF:TGIF(IL-10) ratio contributing to POAG severity. Therefore, it is important that this study has clearly identified, for the first time, in clinical setting the frequency and distribution of blood B-cell subsets as a severity-related signature in a POAG Egyptian patients’ cohort.

Huang et al., investigated POAG eyes and the mean VF defect (MD) of <12 and MD of ≥12 and reported that higher serum IL-4 and BSF-2(IL-6) levels were associated with more advanced VF defects [40]. BSF-2(IL-6) is profibrotic and induces TGF-β, pseudo-exfoliation materials leading to an elevated IOP (mmHg) to disrupt the outflow facility [41] during the way-to-sight loss. B cells’ overproduction of BSF-2(IL-6) stimulates inflammation via producing more pathogenic antibodies with an increase in harming T cells growth [42]. Freedman and Iserovich’s studies reported that intraocular cytokine levels were positively correlated with IOP (mmHg) elevation [43] influencing the aqueous humor dynamics [44] with more IOP (mmHg) increase. 

Currently, the levels of CSIF:TGIF(IL-10) were negatively correlated with POAG MD of VF; however, this correlation did not reach statistical significance. Surprisingly, CSIF:TGIF(IL-10) levels were higher among patients with severe glaucoma compared with patients having mild-to-moderate glaucoma, although this variation was not statistically significant. B-cell subsets that produces CSIF:TGIF(IL-10) are protective and slow the inflammatory diseases’ course [45,46].

To our knowledge, we are the first to perform ROC curve analysis for B cells and their subsets in POAG patients (study strength). From ROC analysis, the total blood B cells and DN B cells exhibited an excellent capability for diagnosing POAG. DN B cells exhibited a significantly better discriminative performance than the BSF-2(IL-6) to CSIF:TGIF(IL-10) ratio in diagnosing and/or predicting POAG disease course or severity. 

B-cell depletion treatment normalizes BSF-2(IL-6) levels produced by multiple sclerosis patients’ B cells, and patients who respond well to B-cell depletion treatment, over an extended period of time, experience a “reset” in their auto-immune condition-related CSIF:TGIF(IL-10) levels’ deficiency, with their re-populated B cells directed towards convenient CSIF:TGIF(IL-10) production [47,48]. This therapeutic strategy can target the expanded B-cell subset, having a good ameliorative impact on disease progression and/or activity. 

Therapeutic strategies to be developed (Tocilizumab an BSF-2(IL-6) receptor antagonist) or repurposed (from in silico findings) include selectively targeting the proinflammatory role of blood B-cell subsets to counteract its cytokine-producing biological function [49,50].

According to the bioinformatics results in Figure 5, BSF-2(IL-6) is one of the blood B-cell surface antigen CVID3(CD19)’s top interacting genes (CD19-IL-6). BSF-2(IL-6) is known to be targeted by ginseng (Drug Bank). 

On the contrary, TNFR(CD27)’s (one of the B-cell surface antigens) top interacting genes is CSIF:TGIF(IL-10) as CD27-IL-10, which mutually affect each other. Consequently, blood B-cell depleted TNFR(CD27) induces the production of INF gamma in T cells (CD27-INFgamma) an axis could be treated by glucosamine (Drug Bank). 

Now, we can suggest that **“POAG Pathogenesis Hallmark(s)”** involves a **“Molecular-Network being Inflammation-Associated”** (in silico retrieved in Figure 4) triggered by an elevated IOP (mmHg) and the systemic low-grade inflammation state as an activating stimulus (BSF-2(IL-6) increased serum level). However, the retinal microenvironment dysregulation was not currently addressed (study limitation, to be conducted shortly).

According to the gene–gene and PPI databases bioinformatics search, the human major immune regulatory cytokine CSIF:TGIF(IL-10) acts on many cells of the immune system, having profound anti-inflammatory functions, limiting excessive tissue disruption coming in via inflammation. Mechanistically, CSIF:TGIF(IL-10) binds to its hetero-tetrameric receptor comprising IL-10RA and IL-10RB leading to JAK1 and STAT2-IL-10 mediated phosphorylation of STAT3. pSTAT3 finds its way to the nucleus, stimulating the anti-inflammatory mediator’s expression to target macrophages and monocytes (they are the antigen-presenting cells) to inhibit BSF-2(IL-6) release and therefore prohibit proinflammation or the acute phase response. This will stop POAG adverse effects from differentiated Ig-secreting cells or lymphocyte and monocyte differentiation.

Recommendation. DN B-cell subset-related genes, effector signaling pathways, and epigenetics identification in silico and clinically in a POAG Egyptian patients’ cohort.

## 5. Summary and Conclusions

Research on abnormalities in B-cell maturation and proliferation/control has provided significant insight into the disease’s pathophysiology and/or clinical management, as the pathophysiology of glaucoma is closely related to immunological and inflammatory blood and B-cell responses. The clinical relevance of measuring and quantifying peripheral blood, as liquid biopsy, B-cell frequency and B-cell subsets distribution as well as circulating pro-/anti-inflammatory cytokines (BSF-2(IL-6), CSIF:TGIF(IL-10), and the BSF-2(IL-6) to CSIF:TGIF(IL-10) ratio) may play an important role in POAG pathogenesis and might help as potential molecular markers to assess POAG disease progression and severity. 

Blocking B-cell surface markers’ target genes, identified via in silico gene–gene interaction network databases and pathway curated databases, would present a promising future potential anti-inflammatory treatment option(s); treatment based on blood B-cell subsets identifying POAG patients; a step-toward compacting blindness increase worldwide. 

## Figures and Tables

**Table 1:** POAG patients (n = 30) and controls (n = 30) demographic characteristics, clinical, and laboratory results.

	Group, n		Significance
Characteristics (Unit)	Cases, 30	Control, 30	*p*-Value
Gender n(%) Male/Female	17(56.7%)/13(43.3%)	16(53.3%)/14(46.7%)	NS
Age (year)	55.5 (49.0–59.2)	51.5 (43.75–55.0)	NS
VA (Log MAR)	0.5 (0.3–0.6)	0.2 (0.00–0.20)	<0.001 *
IOP (mmHg)	18.0 (15.7–22.2)	12.0 (11.0–14.2)	<0.001 *
C/D	0.60 (0.51–0.8)	0.30 (0.20–0.40)	<0.001 *
MD (dB)	−13.0 (−20.3–−5.8)	−2.0 (−2.6–−1.67)	<0.001 *
PSD (dB)	5.6 (3.25–8.7)	2.1 (1.5–2.7)	<0.001 *
WBCs (10^3^/μL)	6.15 (4.6–7.7)	5.7 (4.8–6.6)	NS
Absolute neutrophils count (10^3^/μL)	3.1 (2.4–4.9)	3.0 (2.09–4.0)	NS
Absolute lymphocytes count (10^3^/μL)	1.95 (1.7–2.5)	2.1 (1.7–2.4)	NS
NLR	1.56 (1.08–2.6)	1.5 (1.1–1.7)	NS
Absolute monocytes (10^3^/μL)	0.52 (0.30–0.8)	0.4 (0.30–0.42)	0.019 *
MLR	0.25 (0.14–0.36)	0.18 (0.14–0.23)	0.018 *
Platelets (10^3^/μL)	247.0 (203.3–305.3)	235.0 (204.7–297.2)	NS
PLR	121.7 (95.1–39.4)	118.0 (98.3–134.3)	NS
Total B cells % (CD19+)	12.8 (9.4–15.1)	4.5 (3.6–5.6)	<0.001 *
DN B cells % (CD19+CD27−IgD−)	16.45 (9.85–19.5)	6.7 (4.7–7.92)	<0.001 *
Naïve B cells % (CD19+CD27−IgD+)	59.9 (53.7–70.3)	32.2 (25.8–42.8)	<0.001 *
Unswitched memory B cells % (CD19+CD27+IgD+)	9.3 (7.95–16.3)	21.7 (17.6–32.5)	<0.001 *
Classical switched memory B cells % (CD19+CD27+IgD−)	22.4 (16.7–29.3)	20.6 (10.5–25.7)	NS
BSF-2(IL-6) (ng/L)	58.4 (41.1–76.6)	38.8(35.9–45.7)	<0.001 *
CSIF:TGIF(IL-10) (ng/L)	73.15 (48.0–101.4)	100.1 (84.8–149.6)	0.001 *
BSF-2(IL-6) to CSIF:TGIF(IL-10) ratio	0.76 (0.58–1.32)	0.36 (0.23–0.48)	<0.001 *

Data are presented as the median (IQR: 1st–3rd quartile), statistics were computed using SPSS software, the Mann–Whitney test was used for non-parametric data, and the Chi-square test (*x*^2^) for qualitative data (dichotomous parameters), presented as the absolute number (%) (gender only). * Statistical significance at *p*-value < 0.05. [NS: non-significant. VA: visual acuity, Log MAR: logarithm of minimal angle of resolution, IOP: intraocular pressure, DN: double negative, C/D: cup disc ratio, MD: mean deviation, dB: decibel, PSD: pattern standard deviation of visual field, WBCs: white blood cells, NLR: neutrophil-to-lymphocyte ratio, MLR: monocyte-to-lymphocyte ratio, PLR, platelet-to-lymphocyte ratio, IL: interleukin].

**Table 2:** Spearman’s correlation *r* of B-cell subsets and inflammation biomarkers measured with the glaucoma severity marker among POAG patients (n = 30).

Variable	MD		BSF-2(IL-6) (ng/L)		CSIF:TGIF(IL-10) (ng/L)		BSF-2(IL-6) to CSIF:TGIF(IL-10) Ratio		Cohen’s *q*/Effect Size
	*r*	*p*-Value	*r*	*p*-Value	*r*	*p*-Value	*r*	*p*-Value	
MD	-	-	0.85	<0.001 *	−0.03	NS	0.684	<0.001 *	0.42/medium
DN B cells % (CD19+CD27−IgD−)	0.876	<0.001 *	0.96	<0.001 *	0.065	NS	0.641	<0.001 *	0.588–1.186/large
naïve B cells (CD19+CD27−IgD+)	−0.29	NS	−0.18	NS	−0.045	NS	−0.13	NS	no or small effect
Unswitched memory B cells % (CD19+CD27+IgD+)	−0.84	<0.001 *	−0.97	<0.001 *	−0.129	NS	−0.61	NS	0.871/large
Classical switched memory B cells % (CD19+CD27+IgD−)	0.146	NS	0.22	NS	−0.175 #	NS	0.353 #	NS	0.546 #/large
Cohen’s *q*/effect size	2.579/large		0.69–4/large		no or small effect		no effect		-

Spearman correlation coefficient (*r*) was calculated using SPSS software. All effect sizes were large or medium for the significant *r*; for NS *r*, no or small effect sizes differences were obtained; # effect size was large for CSIF:TGIF(IL-10) and the ratio. * Statistical significance *p*-value < 0.05. Cohen’s *q* effect size estimation on the differences between correlations [NS: non-significant, IL: interleukin, DN: double negative, MD: mean deviation].

**Table 3:** Cut-off values for the discriminative ability of B-cell subsets, inflammation biomarkers to differentiate POAG patients (n = 30) from controls (n = 30) with their AUC, sensitivities, and specificities obtained from ROC curve analysis.

			%			95% C.I.	
Variable	Cut-Off	AUC	SN	SP	*p*-Value	Lower	Upper
Total B cells % (CD19+)	>7.6	0.997	96.7	100	<0.001 *	0.989	1.005
DN B cells % (CD19+CD27−IgD−)	>8.15	0.994	90.0	83.3	<0.001 *	0.889	0.997
Naïve B cells % (CD19+CD27−IgD+)	>44.1	0.951	90.0	83.3	<0.001 *	0.904	0.998
Unswitched memory B cells % (CD19+CD27+IgD+)	<14.45	0.855	73.3	90.0	<0.001 *	0.049	0.241
BSF-2(IL-6) (ng/L)	>47.0	0.805	73.3	83.3	<0.001 *	0.683	0.927
CSIF:TGIF(IL-10) (ng/L)	<87.8	0.754	73.3	73.3	0.001 *	0.630	0.878
BSF-2(IL-6) to CSIF:TGIF(IL-10) ratio	>0.55	0.859	80.0	90.0	<0.001 *	0.763	0.956

Data obtained from the ROC curve analysis using SPSS software. * Statistically significant *p*-value < 0.05, asymptomatic 95% C.I. expressed as (lower bound–upper bound). [AUC: area under the curve, SN: sensitivity, SP: specificity, IL: interleukin, DN: double negative, C.I.: confidence interval].

**Table 4:** Distribution of B-cell subsets and inflammation biomarkers according to glaucoma severity in POAG patients (n = 30).

	POAG Group (n = 30) Subclass, n		Significance
Characteristics (Unit)	Mild-to-Moderate, 12	Severe, 18	*p*-Value
Total B cells % (CD19+)	12.7 (9.5–14.7)	12.8 (9.12–15.3)	NS
DN B cells % (CD19+CD27−IgD−)	9.0 (8.12–10.4)	18.9 (16.9–22.9)	<0.001 *
Naïve B cells % (CD19+CD27−IgD+)	65.9 (56.27–76.6)	58.8 (44.8–68.2)	NS
Unswitched memory B cells % (CD19+CD27+IgD+)	18.6 (13.5–24.8)	8.3 (6.6–9.07)	<0.001 *
Classical switched memory B cells % (CD19+CD27+IgD−)	22.2 (11.6–27.7)	22.9 (17.4–36.6)	NS
BSF-2(IL-6) (ng/L)	38.55 (29.02–50.6)	73.2 (61.1–80.8)	<0.001 *
CSIF:TGIF(IL-10) (ng/L)	71.7 (59.15–104.2)	76.7 (45.4–90.9)	NS
BSF-2(IL-6) to CSIF:TGIF(IL-10) ratio	0.54 (0.35–0.68)	1.0 (0.75–1.7)	<0.001 *

Data are presented as the median (IQR: 1st–3rd quartile), statistics were computed using SPSS software, and the Mann–Whitney test was used for non-parametric data. * Statistical significance at *p*-value < 0.05. [NS: non-significant, IL: interleukin, DN: double negative].

## Data Availability

The original contributions presented in the study are included in the manuscript. Further inquiries can be provided by the corresponding author upon request.

## References

[1] Sarossy M., Crowston J., Kumar D., Weymouth A., Wu Z. (2021). Prediction of glaucoma severity using parameters from the electroretinogram. Sci. Rep..

[2] Nitta K., Tachibana G., Wajima R., Inoue S., Ohigashi T., Otsuka N., Kurashima H., Santo K., Hashimoto M., Shibahara H. (2020). Predicting Lifetime Transition Risk of Severe Visual Field Defects Using Monte Carlo Simulation in Japanese Patients with Primary Open-Angle Glaucoma. Clin. Ophthalmol..

[3] Shin Y.J., Kim E., Han B.K., Yi K. (2020). Serum biomarkers for the diagnosis of glaucoma. Diagnostics.

[4] Zimprich L., Diedrich J., Bleeker A., Schweitzer J.A. (2020). Corneal hysteresis as a biomarker of glaucoma: Current insights. Clin. Ophthalmol..

[5] Tapply I.H., Bourne R.R. (2023). Epidemiology of glaucoma. The Science of Glaucoma Management.

[6] Dammak A., Sanchez Naves J., Huete-Toral F., Carracedo G. (2023). New Biomarker Combination Related to Oxidative Stress and Inflammation in Primary Open-Angle Glaucoma. Life.

[7] Yu L., Chen Y., Xu X., Dong Q., Xiu W., Chen Q., Wang J., He C., Ye J., Lu F. (2021). Alterations in peripheral B cell subsets correlate with the disease severity of human glaucoma. J. Inflamm. Res..

[8] Li Y., Li Z., Hu F. (2021). Double-negative (DN) B cells: An under-recognized effector memory B cell subset in autoimmunity. Clin. Exp. Immunol..

[9] Maity P.C., Datta M., Nicolò A., Jumaa H. (2018). Isotype specific assembly of B cell antigen receptors and synergism with chemokine receptor CXCR4. Front. Immunol..

[10] Inoue-Mochita M., Inoue T., Kojima S., Futakuchi A., Fujimoto T., Sato-Ohira S., Tsutsumi U., Tanihara H. (2018). Interleukin-6–mediated trans-signaling inhibits transforming growth factor-β signaling in trabecular meshwork cells. J. Biol. Chem..

[11] Ulhaq Z.S., Soraya G.V., Hasan Y.T., Rachma L.N., Rachmawati E., Shodry S., Kusuma M.A. (2022). Serum IL-6/IL-10 ratio as a biomarker for the diagnosis and severity assessment of primary-open angle glaucoma. Eur. J. Ophthalmol..

[12] Zhang N., Wang J., Li Y., Jiang B. (2021). Prevalence of primary open angle glaucoma in the last 20 years: A meta-analysis and systematic review. Sci. Rep..

[13] Spaeth G.L. (2021). European Glaucoma Society Terminology and Guidelines for Glaucoma. Br. J. Ophthalmol..

[14] Shi X., Yu Z., Ren P., Dong X., Ding X., Song J., Zhang J., Li T., Wang C. (2023). HUSCH: An integrated single-cell transcriptome atlas for human tissue gene expression visualization and analyses. Nucleic Acids Res..

[15] Dimitrakopoulos G.N., Klapa M.I., Moschonas N.K. (2022). How Far Are We from the Completion of the Human Protein Interactome Reconstruction?. Biomolecules.

[16] Fernandes J.D., Hinrichs A.S., Clawson H., Gonzalez J.N., Lee B.T., Nassar L.R., Raney B.J., Rosenbloom K.R., Nerli S., Rao A.A. (2020). The UCSC SARS-CoV-2 genome browser. Nat. Genet..

[17] Lenhard W., Lenhard A. (2022). Computation of Effect Sizes. https://www.psychometrica.de/effect_size.html.

[18] Williams P.A., Braine C.E., Kizhatil K., Foxworth N.E., Tolman N.G., Harder J.M., Scott R.A., Sousa G.L., Panitch A., Howell G.R. (2019). Inhibition of monocyte-like cell extravasation protects from neurodegeneration in DBA/2J glaucoma. Mol. Neurodegener..

[19] Wei X., Cho K.S., Thee E.F., Jager M.J., Chen D.F. (2019). Neuroinflammation and microglia in glaucoma: Time for a paradigm shift. J. Neurosci. Res..

[20] Vázquez-Mendoza A., Vannan D., Morales E.G., González M.I., Hernández J.L. (2021). Lymphocytes in Dry Eye Disease. Dry Eye Syndrome-Modern Diagnostic Techniques and Advanced Treatments.

[21] Tong Y., Zhou Y.L., Zheng Y., Biswal M., Zhao P.Q., Wang Z.Y. (2017). Analyzing cytokines as biomarkers to evaluate severity of glaucoma. Int. J. Ophthalmol..

[22] Shestopalov V.I., Spurlock M., Gramlich O.W., Kuehn M.H. (2021). Immune responses in the glaucomatous retina: Regulation and dynamics. Cells.

[23] Chen H., Cho K.S., Vu T.K., Shen C.H., Kaur M., Chen G., Mathew R., McHam M.L., Fazelat A., Lashkari K. (2018). Commensal microflora-induced T cell responses mediate progressive neurodegeneration in glaucoma. Nat. Commun..

[24] Claes N., Fraussen J., Vanheusden M., Hellings N., Stinissen P., Van Wijmeersch B., Hupperts R., Somers V. (2016). Age-associated B cells with proinflammatory characteristics are expanded in a proportion of multiple sclerosis patients. J. Immunol..

[25] Sachinidis A., Garyfallos A. (2021). Double Negative (DN) B cells: A connecting bridge between rheumatic diseases and COVID-19?. Mediterr. J. Rheumatol..

[26] Ruschil C., Gabernet G., Lepennetier G., Heumos S., Kaminski M., Hracsko Z., Irmler M., Beckers J., Ziemann U., Nahnsen S. (2020). Specific induction of double negative B cells during protective and pathogenic immune responses. Front. Immunol..

[27] Wei C., Anolik J., Cappione A., Zheng B., Pugh-Bernard A., Brooks J., Lee E.H., Milner E.C., Sanz I. (2007). A new population of cells lacking expression of CD27 represents a notable component of the B cell memory compartment in systemic lupus erythematosus. J. Immunol..

[28] Jenks S.A., Cashman K.S., Zumaquero E., Marigorta U.M., Patel A.V., Wang X., Tomar D., Woodruff M.C., Simon Z., Bugrovsky R. (2018). Distinct effector B cells induced by unregulated toll-like receptor 7 contribute to pathogenic responses in systemic lupus erythematosus. Immunity.

[29] Moysidou E., Lioulios G., Christodoulou M., Xochelli A., Stai S., Iosifidou M., Iosifidou A., Briza S., Briza D.I., Fylaktou A. (2023). Increase in Double Negative B Lymphocytes in Patients with Systemic Lupus Erythematosus in Remission and Their Correlation with Early Differentiated T Lymphocyte Subpopulations. Curr. Issues Mol. Biol..

[30] Wu Y.C., Kipling D., Dunn-Walters D.K. (2011). The relationship between CD27 negative and positive B cell populations in human peripheral blood. Front. Immunol..

[31] De Gruijter N.M., Jebson B., Rosser E.C. (2022). Cytokine production by human B cells: Role in health and autoimmune disease. Clin. Exp. Immunol..

[32] Rutigliani C., Tribble J.R., Hagström A., Lardner E., Jóhannesson G., Stålhammar G., Williams P.A. (2022). Widespread retina and optic nerve neuroinflammation in enucleated eyes from glaucoma patients. Acta Neuropathol. Commun..

[33] Yang X., Zeng Q., Göktaş E., Gopal K., Al-Aswad L., Blumberg D.M., Cioffi G.A., Liebmann J.M., Tezel G. (2019). T-lymphocyte subset distribution and activity in patients with glaucoma. Investig. Ophthalmol. Vis. Sci..

[34] Irkec M.T., Bozkurt B., Mesci L., Bulur B., Ersoy F., Sanal O., Orhan M., Arslan U., Tezcan I. (2005). TNF–alpha and IL–10 gene polymorphisms in Turkish glaucoma patients. Investig. Ophthalmol. Vis. Sci..

[35] Chua J., Vania M., Cheung C.M., Ang M., Chee S.P., Yang H., Li J., Wong T.T. (2012). Expression profile of inflammatory cytokines in aqueous from glaucomatous eyes. Mol. Vis..

[36] Gramlich O.W., Beck S., von Thun und Hohenstein-Blaul N., Boehm N., Ziegler A., Vetter J.M., Pfeiffer N., Grus F.H. (2013). Enhanced insight into the autoimmune component of glaucoma: IgG autoantibody accumulation and pro-inflammatory conditions in human glaucomatous retina. PLoS ONE.

[37] Duddy M.E., Alter A., Bar-Or A. (2004). Distinct profiles of human B cell effector cytokines: A role in immune regulation?. J. Immunol..

[38] Borkenstein A., Faschinger C., Maier R., Weger M., Theisl A., Demel U., Graninger W., Irene H., Mossböck G. (2013). Measurement of tumor necrosis factor-alpha, interleukin-6, Fas ligand, interleukin-1α, and interleukin-1β in the aqueous humor of patients with open angle glaucoma using multiplex bead analysis. Mol. Vis..

[39] Takai Y., Tanito M., Ohira A. (2012). Multiplex cytokine analysis of aqueous humor in eyes with primary open-angle glaucoma, exfoliation glaucoma, and cataract. Investig. Ophthalmol. Vis. Sci..

[40] Huang P., Qi Y., Xu Y.S., Liu J., Liao D., Zhang S.S., Zhang C. (2010). Serum cytokine alteration is associated with optic neuropathy in human primary open angle glaucoma. J. Glaucoma.

[41] Zenkel M., Lewczuk P., Jünemann A., Kruse F.E., Naumann G.O., Schlötzer-Schrehardt U. (2010). Proinflammatory cytokines are involved in the initiation of the abnormal matrix process in pseudoexfoliation syndrome/glaucoma. Am. J. Pathol..

[42] Vazquez M.I., Catalan-Dibene J., Zlotnik A. (2015). B cells responses and cytokine production are regulated by their immune microenvironment. Cytokine.

[43] Freedman J., Iserovich P. (2013). Pro-inflammatory cytokines in glaucomatous aqueous and encysted Molteno implant blebs and their relationship to pressure. Investig. Ophthalmol. Vis. Sci..

[44] Huang W., Chen S., Gao X., Yang M., Zhang J., Li X., Wang W., Zhou M., Zhang X., Zhang X. (2014). Inflammation-related cytokines of aqueous humor in acute primary angle-closure eyes. Investig. Ophthalmol. Vis. Sci..

[45] Mizoguchi A., Mizoguchi E., Takedatsu H., Blumberg R.S., Bhan A.K. (2002). Chronic intestinal inflammatory condition generates IL-10-producing regulatory B cell subset characterized by CD1d upregulation. Immunity.

[46] Mauri C., Gray D., Mushtaq N., Londei M. (2003). Prevention of arthritis by interleukin 10–producing B cells. J. Exp. Med..

[47] Barr T.A., Shen P., Brown S., Lampropoulou V., Roch T., Lawrie S., Fan B., O’Connor R.A., Anderton S.M., Bar-Or A. (2012). B cell depletion therapy ameliorates autoimmune disease through ablation of IL-6–producing B cells. J. Exp. Med..

[48] Menon M., Blair P.A., Isenberg D.A., Mauri C. (2016). A regulatory feedback between plasmacytoid dendritic cells and regulatory B cells is aberrant in systemic lupus erythematosus. Immunity.

[49] Moura R.A., Quaresma C., Vieira A.R., Goncalves M.J., Polido-Pereira J., Romao V.C., Martins N., Canhao H., Fonseca J.E. (2017). B-cell phenotype and IgD-CD27-memory B cells are affected by TNF-inhibitors and tocilizumab treatment in rheumatoid arthritis. PLoS ONE.

[50] Han S., Zhuang H., Xu Y., Lee P., Li Y., Wilson J.C., Vidal O., Choi H.S., Sun Y., Yang L.J. (2015). Maintenance of autoantibody production in pristane-induced murine lupus. Arthritis Res. Ther..

